# The Berlin-Buch respiration chamber for energy expenditure measurements

**DOI:** 10.1007/s00421-023-05164-w

**Published:** 2023-02-27

**Authors:** Anja Mähler, Till Schütte, Jochen Steiniger, Michael Boschmann

**Affiliations:** 1 Experimental and Clinical Research Center, a cooperation between the Max Delbrück Center in the Helmholtz Association and Charité - Universitätsmedizin Berlin, Lindenberger Weg 80, 13125 Berlin, Germany; 2 Charité – Universitätsmedizin Berlin, corporate member of Freie Universität Berlin and Humboldt-Universität zu Berlin, Lindenberger Weg 80, 13125 Berlin, Germany; 3 Max Delbrück Center in the Helmholtz Association (MDC), Berlin, Germany; 4 Berlin Institute of Health at Charité – Universitätsmedizin Berlin, Clinical Study Center (CSC), Charitéplatz 1, 10117 Berlin, Germany

**Keywords:** Whole-room calorimeter, Respiration chamber, Energy expenditure, Diet-induced thermogenesis

## Abstract

**Purpose:**

We present a methodological overview of a respiration chamber at the Experimental and Clinical Research Center in Berlin, Germany. Since 2010, we investigated 750 healthy subjects and patients with various diseases. We routinely measure resting energy expenditure (REE), dietary-induced thermogenesis, and activity energy expenditure.

**Methods:**

The chamber is a pull calorimeter with a total volume of 11,000 L. The majority of measurements is done with a flow rate of 120 L/min, yielding a favorable time constant of 1.53 h. The gas analysis system consists of two paramagnetic O_2_ sensors and two infrared CO_2_ sensors, one for incoming and one for outgoing air samples. O_2_ and CO_2_ sensors are calibrated simultaneously before each measurement with a 6 min calibration routine. To verify the accuracy of the whole the calorimetric system, it is validated every 2 weeks by 2 h acetone burning tests.

**Results:**

Validation factors (calculated/measured) of 20 representative 2 h acetone burning tests were 1.03 ± 0.03 for $\dot{V}{\text{O}}_{2}$, 1.02 ± 0.02 for $\dot{V}{\text{CO}}_{{2}}$, 0.99 ± 0.02 for RER, and 1.03 ± 0.03 for EE. Four repeated 60 min REE measurements of a healthy woman showed variabilities of 231.9 ± 4.8 ml/min for $\dot{V}{\text{O}}_{2}$ (CV 2.1%), 166.0 ± 6.3 ml/min for $\dot{V}{\text{CO}}_{{2}}$ (CV 3.8%), 0.73 ± 0.03 for RER (CV 4.6%), and 4.55 ± 0.07 kJ/min for EE (CV 1.6%).

**Conclusions:**

The data presented show that our respiration chamber produces precise and valid EE measurements with an exceptionally fast responsiveness.

**Graphical Abstract:**

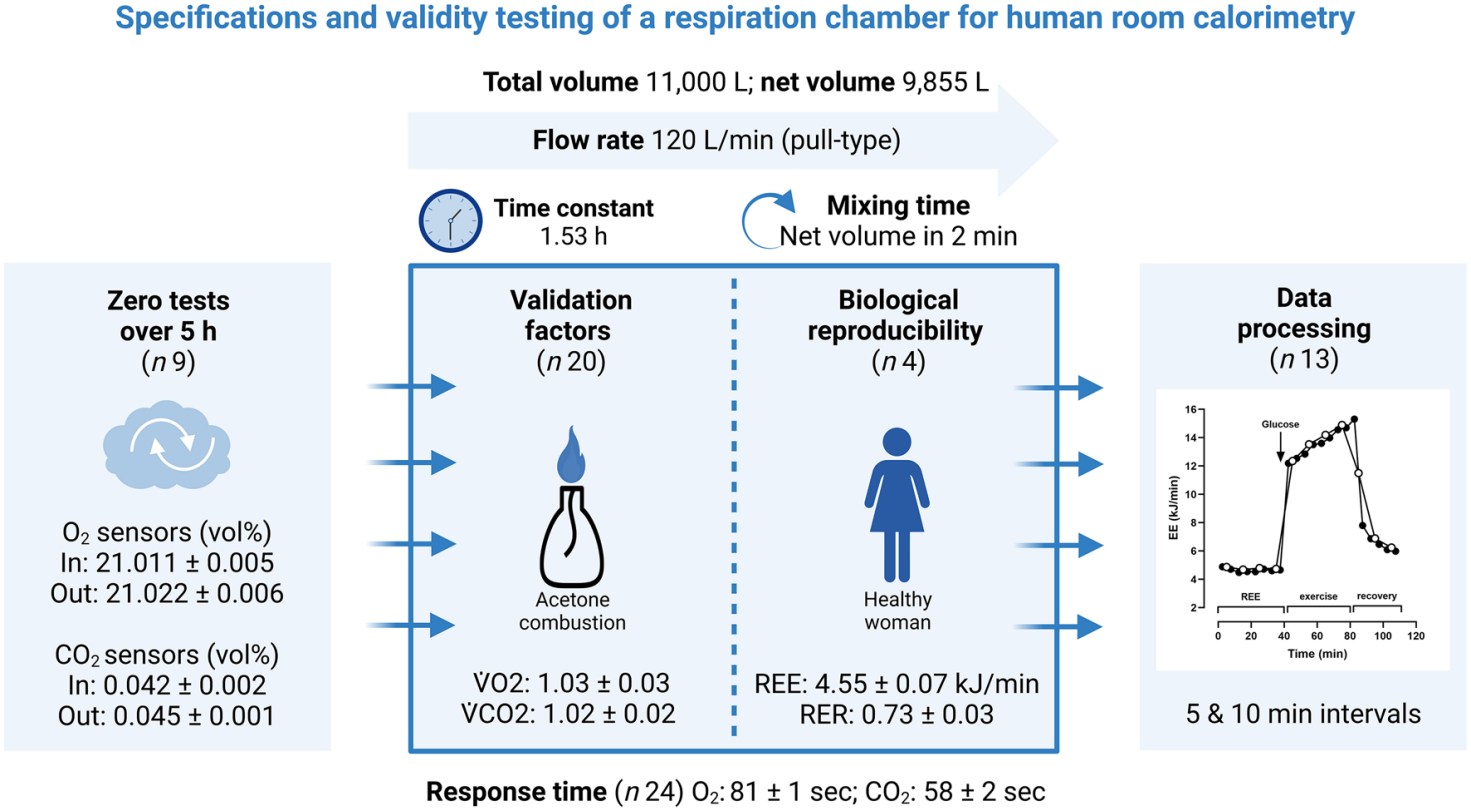

## Introduction

All life processes require energy, and these energy costs can be estimated by calorimetry, either directly by measuring heat production or indirectly by measuring metabolic by-products (McLean and Tobin 2008). The predominantly method used today is indirect calorimetry, i.e., measurement of oxygen consumption ($\dot{V}{\text{O}}_{2}$), carbon dioxide production ($\dot{V}{\text{CO}}_{{2}}$), and urinary nitrogen excretion. Energy expenditure (EE) and macronutrient oxidation are calculated from these by-products of energy metabolism by empirically derived equations.

There are three systems available for calorimetric measurements – facemasks, ventilated hood systems and whole-room calorimeters/respiration chambers. Ventilated hoods are convenient for short protocols, such as resting EE (REE) measurements. Respiration chambers allow for longer measurements, which closer resemble free-living conditions.

In 1866, Pettenkofer and Voit introduced the first whole-body indirect calorimeter for humans. By minutely detailed measurements of body weight, food composition, $\dot{V}{\text{O}}_{2}$, $\dot{V}{\text{CO}}_{{2}}$, water evaporation, and urine and stool energy content, they showed that not mere elements, but kind and amount of ingested macronutrients determine EE (Pettenkofer and Voit 1866).

Another significant landmark in human calorimetry was the combined direct and indirect respiration chamber by Atwater and Benedict. They introduced an open-circuit system in 1899 and a closed-circuit system 1905 (Atwater and Benedict 1905), with which they could accurately measure heat production, $\dot{V}{\text{O}}_{2}$ and $\dot{V}{\text{CO}}_{{2}}$. Using this combined system, they could show the close correlation between direct and indirect calorimetric measurements. However, these measurements were quite expensive and technically laborious (McLean and Tobin 2008).

In time technically advanced gas analyzers based on physical principles were implemented into calorimetric systems leading to higher accuracy of gaseous exchange measurements. More recently, data acquisition systems and computing power facilitated the development of modern respiration chambers with high accuracy and validity. In addition to technical advancements, theoretical understanding of indirect calorimetry as well as EE and macronutrient oxidation equations expanded our knowledge of the dynamics of central metabolic processes.

Here, we present a thorough methodological overview of our respiration chamber established in 2010. We elaborate on design, calibration and validation routines, time constant, and study protocol application.

## Methods

### Design and layout

The respiration chamber at the Experimental and Clinical Research Center in Berlin-Buch, Germany was established in 2010. This was an interdisciplinary team effort based on over 30 years of scientific experience with indirect calorimetry (Steiniger 1984, 1985; Steiniger et al. 1987; Boschmann et al. 2007). Technical expertise was provided by the main contractor Linde GmbH (Gases Division, Berlin, Germany) and by subcontractors for dry construction, ventilation (LORMS Service AG, Ahrensfelde, Germany), measurement and control technology (digitech gmbh, Ahrensfelde, Germany) and for sensors and gas analysis (HTK Hamburg GmbH, Germany).

The chamber (length 2.5 m, width 2.0 m, height 2.2 m, total volume 11,000 L, net volume 9900 L) is a room-in-room dry construction. Thermal insulation and ventilation system are integrated between the inner and outer chamber walls. To prevent air leaks, the inner walls are sealed with a special paint (CreaGlas 2 K-PU-Finish 3471, Brillux GmbH & Co. KG, Münster, Germany). High precision sensors measure oxygen, carbon dioxide, airflow, and climatic conditions. An individual and flexible self-programmed software package based on Microsoft Visual Studio controls measurements, collects, and processes all data required to assess changes in EE and macronutrient oxidation. Raw data are transferred into several MS Excel files for further analysis.

**Fig. 1:**
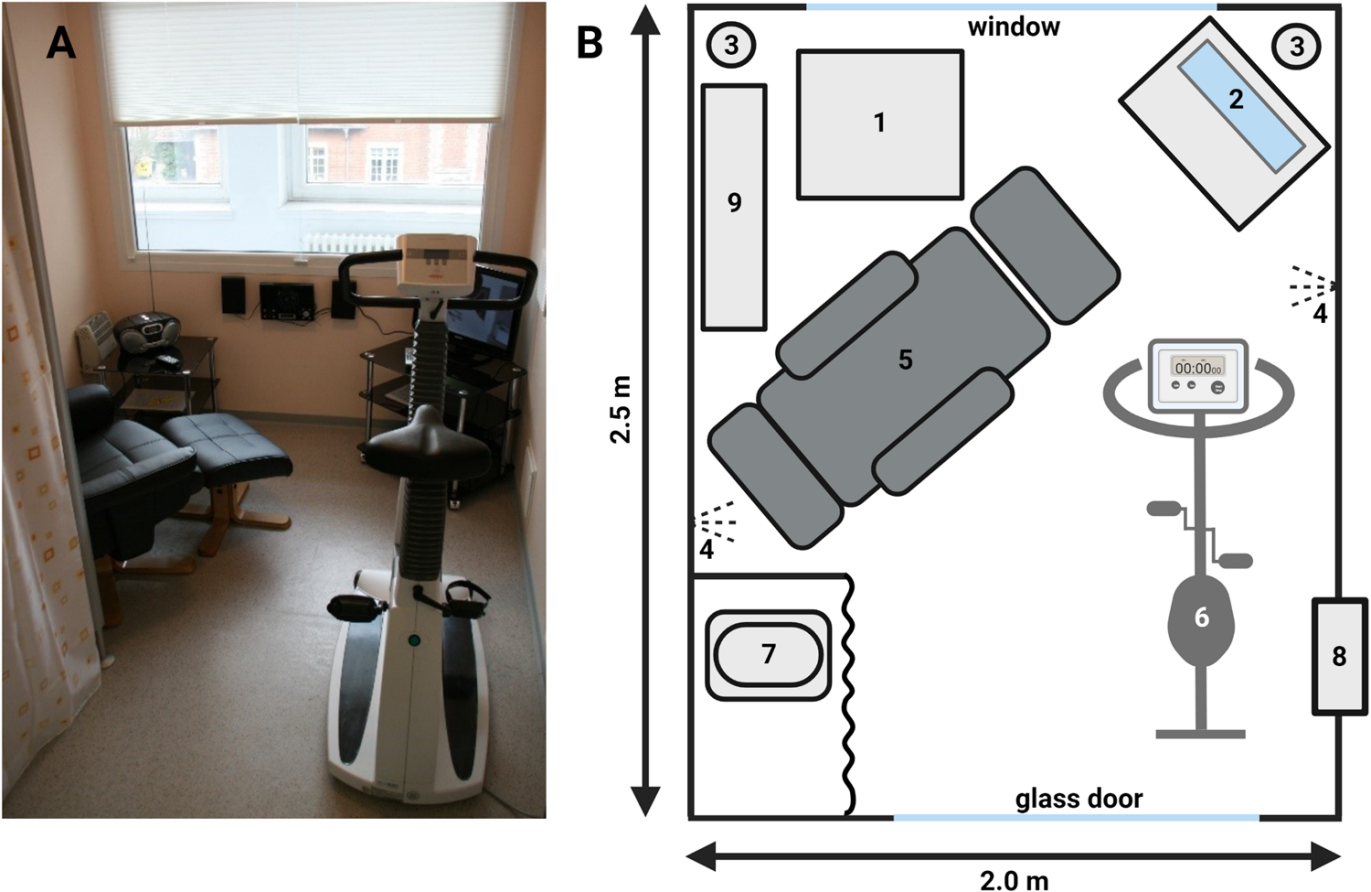
Photograph (**A**) and layout (**B**) of the pull-type, open-circuit respiration chamber in Berlin, Germany. 1, table; 2, television set; 3, cameras; 4, Passive Infrared Sensors; 5, chair with footrest; 6, bicycle ergometer; 7, toilet; 8, air lock; 9, air conditioning

The chamber has a great airtight window and a glass door allowing subjects to look outside (Fig. 1A). Both window and door can be covered with blinds from inside the chamber for privacy. Technical equipment is located outside the chamber to create a non-stressful environment. The chamber is equipped with air conditioning, a comfortable chair with a footrest, a table, TV rack, TV/DVD set, a bicycle ergometer, and a camping toilet (Fig. 1B). Bicycle ergometer and chair can be replaced by a bed for sleeping EE measurements. Therefore, the set-up allows measuring EE from 30 min up to 24 h.

Close supervision of the subject is facilitated by two mini colour cameras, one pointing to the armchair, one to the bicycle ergometer. Of note, there are no recordings at any time. If necessary, subject and investigator can communicate via an intercom. Camera supervision can be discontinued if requested by the subject.

The chamber is equipped with three passive infrared sensors (PIRS), one in front and two above the subject. PIRS react to heat emanating from moving bodies, i.e. humans and animals. If the module recognizes a movement, it produces a digital signal. The signal is registered in volt, thus allowing for semi-quantitative evaluation of activity.

The quite air conditioning (Sanyo SAP-FDRV96EH, 3 kW, 300–500 m^3^/h, 22–30 dB) can be adjusted by the subject from within or by the investigator from outside.

### Flow rate and sensors

Fresh air is pulled into the chamber at four entry points near the ceiling, is mixed by the air conditioning, and exits at four points in the middle of the opposite wall (pull calorimeter). This air flow is facilitated by a precision pump (ORPU V01Y, Pumpenfabrik GmbH, Germany) and can be adjusted to a flow rate between 100 and 200 L/min. Flow is measured by a thermal mass flow controller (HTK Hamburg, Germany). The majority of measurements is done with a flow rate of 120 L/min. The negative pressure registered by the chamber at this flow rate is about 6 kPa.

Gas analysis is controlled by a Process Control Unit (PCU10-O_2_/CO_2_-S, HTK Hamburg, Germany). The unit contains two paramagnetic O_2_ sensors (range 20.000–21.000 Vol%, deficit 0.000–1.000 Vol%, HUMMINGBIRD, Servomex, UK) and two NIDR infrared CO_2_ sensors (0.000–1.000 Vol%, Dynament Ltd, UK), one for incoming and one for outgoing air samples. Resolution of all four sensors is better than 0.001 Vol%. Two diaphragm pumps draw aliquot samples (80 mL/min) of incoming and outgoing air to the respective gas analysis system.

Every second, flow rate, barometric pressure, temperature, relative humidity, and PIRS measurements are digitalized (DT9813-10 V, 12 bit, Data Translation Inc.) and transferred to the computer (RS 232, USB 2.0). The interface was programmed using the DT-Open Layers for NET Class Library (Data Translation Inc.). Further analysis is done with 1 min averages of these data with no further signal processing techniques.

### Calibration

Before each measurement, O_2_ and CO_2_ sensors are calibrated simultaneously with three calibration gases: 20.0% O_2_ in N_2_, 21.0 O_2_ in N_2_ and 0.8% CO_2_ in N_2_. Certified accuracy is ± 0.2% for O_2_ and ± 0.02% for CO_2_ (Linde GmbH, gases division, Berlin, Germany). Gases enter the gas analysis system at a flow rate of 80 mL/min. The software triggers a 360 s calibration routine. Valves that open and close to allow calibration gas flow switch at 0 s (O_2_ zero, 20%), 120 s (O_2_ span, 21% and CO_2_ zero, 0%), 240 s (CO_2_ span, 0.803%), and 360 s (end). Calibration analysis is based on data collection during concentration plateaus after 120 s for O_2_ sensors and after 100 s for CO_2_ sensors. All calibration data are saved in MS Excel files to monitor precision and stability of all gas sensors over time.

### Validation

To verify the accuracy of $\dot{V}{\text{O}}_{2}$, $\dot{V}{\text{CO}}_{{2}}$, EE, and respiratory exchange ratio (RER) measurements, the calorimetric system is validated every 2 weeks by 2 h acetone burning tests. For this, a wick lamp filled with acetone (purity > 99%) is placed on a digital scale and ignited. The scale (maximal load 1000 g, resolution 0.01 g, KERN PCB 1000–2, Kern & Sohn GmbH, Germany) is connected to the computer by a RS 232 interface in order to register the amount of burned acetone.

Theoretically, burning of 1 g acetone (corrected for acetone content of 99.95% and evaporation of 0.0066 g/min (own data)) consumes 1.545 L O_2_ and produces 1.150 L CO_2_. Consequently, RER ($\dot{V}{\text{CO}}_{{2}}$/$\dot{V}{\text{O}}_{2}$) and EE should be 0.746 and 30.42 kJ, respectively. Calculated values are divided by measured values during the validation to receive validation factors (calculated/measured). The factors for $\dot{V}{\text{O}}_{2}$ and $\dot{V}{\text{CO}}_{{2}}$ are used to correct subject measurements within the respective validation interval (usually 2 weeks).

In addition to regular 2 h validations, we do 20 h measurements that consist of three phases: an acetone burning phase for validation purposes (~ 8 h), a washout phase to determine chamber volume (~ 7 h), and a comparison phase to evaluate sensor stability over time (~ 5 h).

### Calculations and algorithms

$\dot{V}{\text{O}}_{2}$ and $\dot{V}{\text{CO}}_{{2}}$ are calculated from gas volumes and climatic data with fixed averaging windows of 5, 10, or 15 min using the equations by Brown et al. (1984). From these volumes, EE is calculated using glucose, fatty acids, and amino acids as reference systems. Stoichiometric factors for carbohydrate oxidation are derived from glucose (C_6_H_12_O_6_) and for fat oxidation from palmitoyl-stearoyl-oleoyl-glycerol (C_22_H_104_O_6_) according to Ferrannini et al. (Ferrannini 1988). For protein, we use the nine amino acids most relevant for energy metabolism (ALA, ASP, ASN, GLU, GLN, LEU, ILE, ARG, LYS; combined formula C_5.0_H_10.6_O_2.7_N_1.7_). We presume that 100% of nitrogen liberated in protein metabolism is excreted as urea via the urine.

1. Glucose (g) = − 3.200 $$\dot{V}{\text{O}}_{2}$$(L) + 4.541 $$\dot{V}{\text{CO}}_{{2}}$$ (L) − 2.688 N_ex_ (g); (15.65 kJ/g)
2. Palmitoyl-stearoyl-oleoyl-glycerol (g) = 1.669 $\dot{V}{\text{O}}_{2}$ (L) − 1.669 $\dot{V}{\text{CO}}_{{2}}$ (L) − 1.414 N_ex_ (g); (39.75 kJ/g)
3. Amino acids (g) = 5.848 N_ex_ (g); (16.14 kJ/g)
4. EE (kJ/min) = 16.28 $\dot{V}{\text{O}}_{2}$ (L) + 4.70 $\dot{V}{\text{CO}}_{{2}}$ (L) − 3.88 N_ex_ (g)
  Under resting conditions and diets low in protein, a simplified formula can be used since nitrogen excretion (N_ex_) is nearly constant at 5.33 mg/min (Steiniger et al. 2009).
5. EE (kJ/min) = 16.07 $\dot{V}{\text{O}}_{2}$ (L/min) + 4.89 $\dot{V}{\text{CO}}_{{2}}$ (L/min) − 0.035

### Statistics

Statistical analyses were performed with GraphPad Prism (Version 9.2.0). Data are given as mean ± SD. Gas sensor variablilty was evaluted by simple linear regression. ROUT method was used to detect outliers with a desired maximum false discovery rate of 1%.

## Results

### Calibration routine

**Fig. 2:**
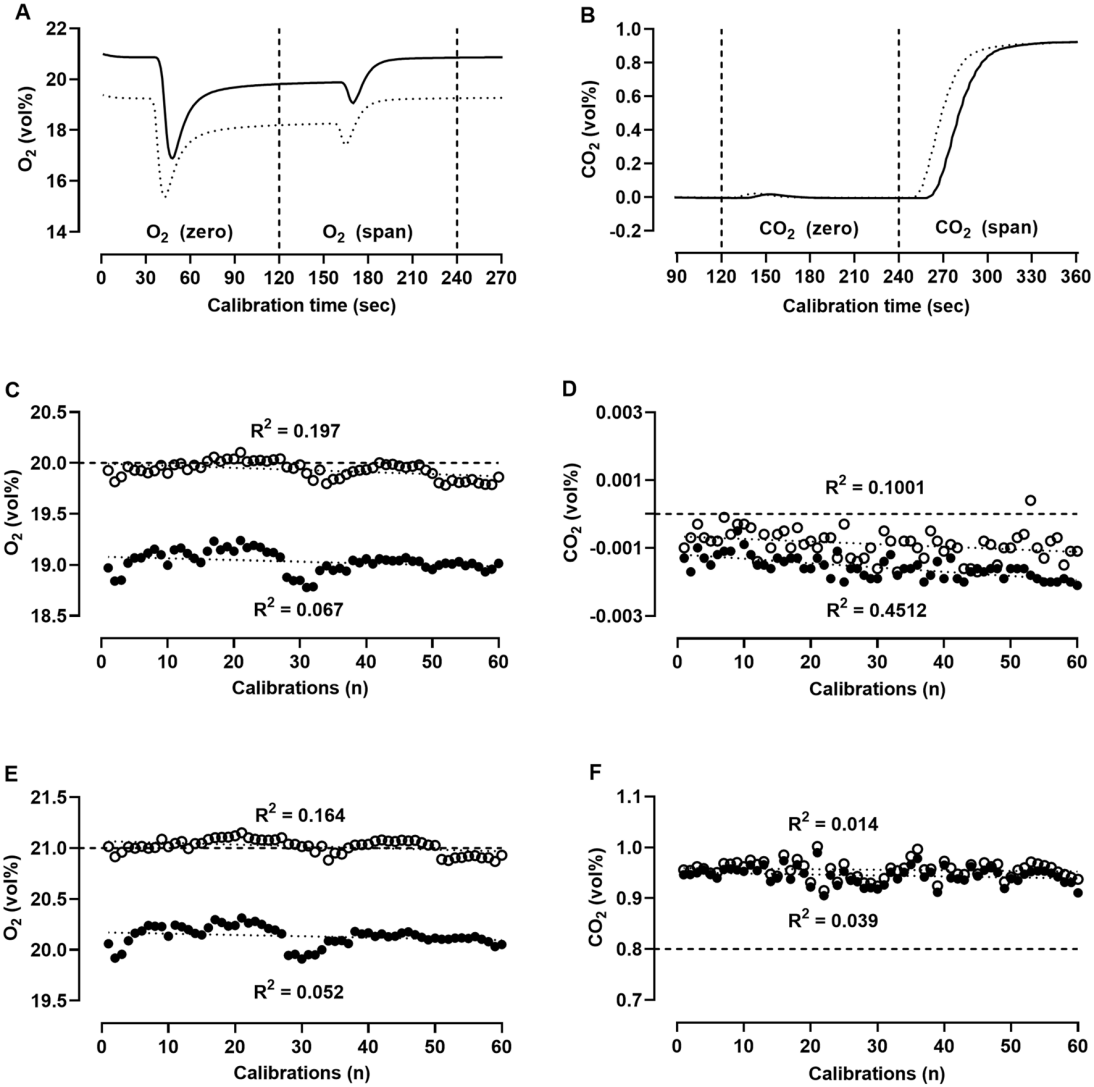
Representative time course of **A** oxygen (O_2_) and **B** carbon dioxide (CO_2_) concentration during an automatic calibration routine; incoming sensors (dotted lines), outgoing sensors (solid lines), valves switch at 0, 120, 240, and 360 s (dashed vertical lines). Results of calibrations (*n* = 60) over 2 years for **C, E** oxygen sensors and **D, F** carbon dioxide sensors; incoming sensors (closed circles), outgoing sensors (open circles), reference gas concentration (dashed lines), and linear regression results (dotted lines). *R*^2^ values by simple linear regression

Figure 2 shows one representative calibration time course and 60 calibrations done over 2 years. Incoming and outgoing O_2_ sensors register O_2_ concentrations with a difference of about 1 vol%. This difference is cancelled out by the calibration equations. Linear regression values for stability of calibration results ranged from 0.039 to 0.451 (Fig. 2), indicating minimal to small sensor drift over a period of 2 years.

### Validation

**Fig. 3:**
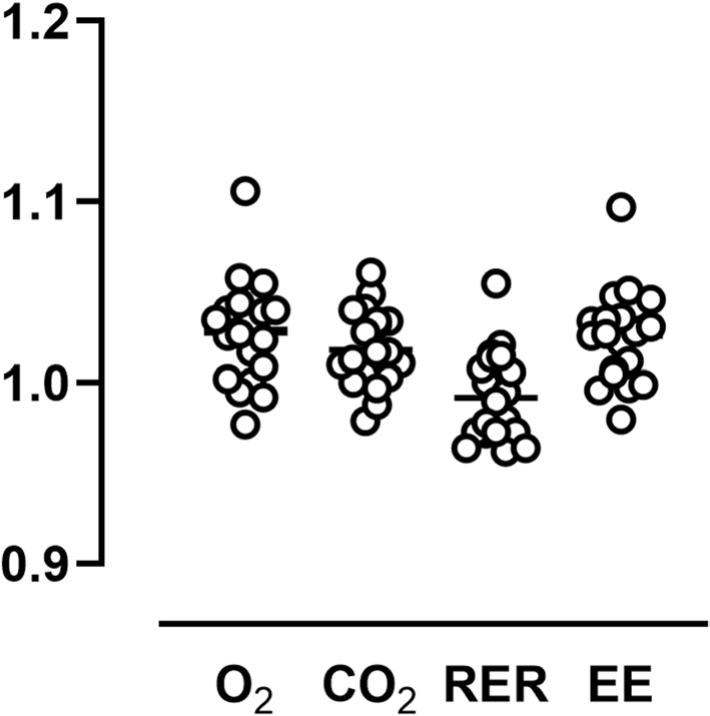
Validation factors (calculated/measured) for $\dot{V}{\text{O}}_{2}$, $\dot{V}{\text{CO}}_{{2}}$, respiratory exchange ratio (RER), and energy expenditure (EE) of 20 2 h acetone burning tests

Validation factors (calculated/measured) of 20 representative 2 h acetone burning tests were 1.03 ± 0.03 for $\dot{V}{\text{O}}_{2}$, 1.02 ± 0.02 for $\dot{V}{\text{CO}}_{{2}}$, 0.99 ± 0.02 for RER, and 1.03 ± 0.03 for EE (Fig. 3).

### Chamber net volume

The washout phases of six 20 h measurements within 2 years (unchanged furnishing) were used to calculate the net volume of the respiration chamber. At a flow rate of 120 L/min and air conditioning power of 300 m^3^/h, volume measurements were 9855 ± 486 L (corresponding to 8629 ± 421 L at standard temperature and pressure, dry (STPD)).

### Gas sensor stability

**Fig. 4:**
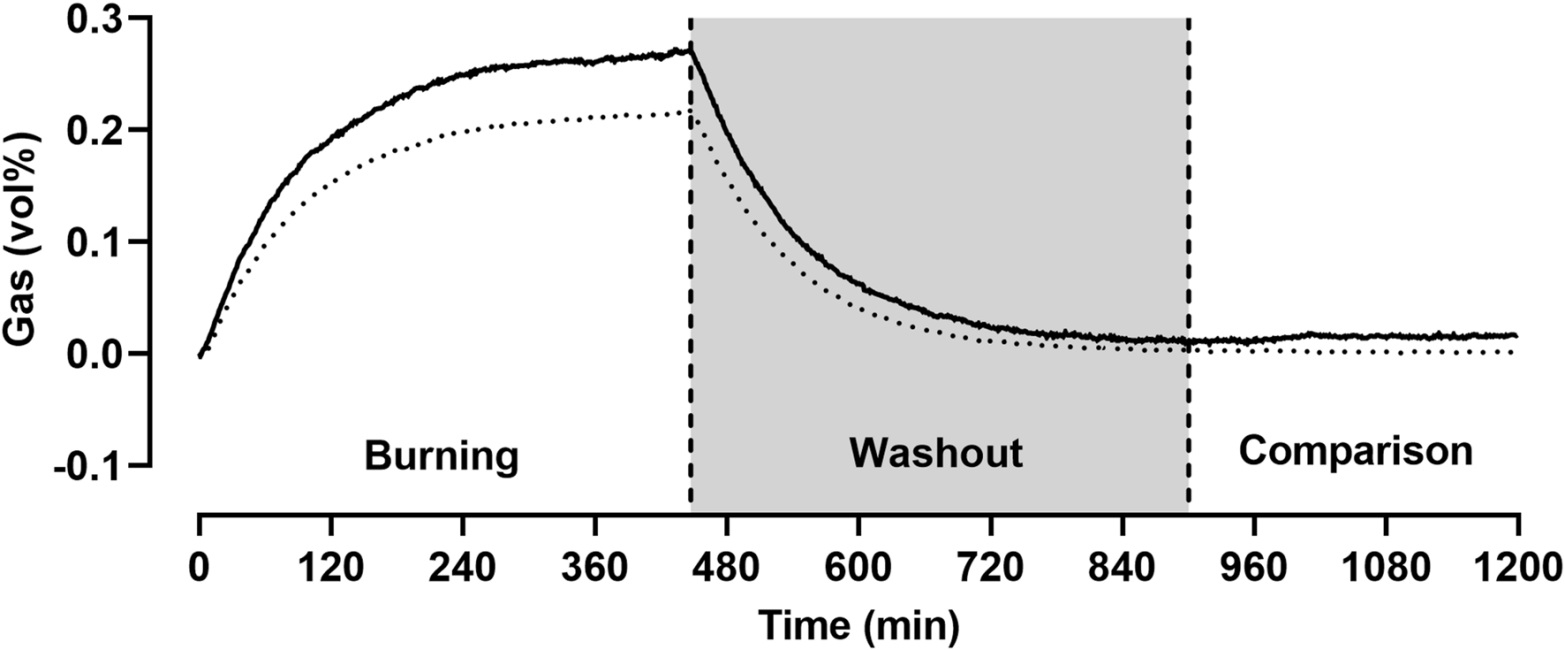
Time course of one representative 20 h validation to determine chamber volume. Oxygen (O_2_) incoming–outgoing (solid line), carbon dioxide (CO_2_) outgoing–incoming (dotted line)

The 5 h comparison phase during 20 h measurements should show comparable values for incoming and outgoing O_2_ and CO_2_ sensors and a stability of each sensor over time (zero test). In one example meaurement (Fig. 4), we recorded O_2_ sensor variabilities of 21.087 ± 0.006 vol% (incoming) and 21.072 ± 0.007 vol% (outgoing). The difference between O_2_ sensors was 0.014 ± 0.002 vol%. CO_2_ sensor variabilities were 0.047 ± 0.0004 vol% (incoming) and 0.049 ± 0.0006 vol% (outgoing); difference 0.018 ± 0.0007 vol%.

Similar results were obtained by nine 20 h measurements within 2 years. Mean variabilities for O_2_ sensors were 21.011 ± 0.005 vol% (incoming) and 21.022 ± 0.006 vol% (outgoing) for CO_2_ sensors 0.042 ± 0.0016 vol% (incoming) and 0.045 ± 0.0007 vol% (outgoing).

### Time constant

The time constant of a respiration chamber is the relation between chamber volume and flow rate. This value ranges from 1 to 8 h in different chambers around the world (Dallosso and James 1984; Dauncey et al. 1978; Henning et al. 1996; Nguyen et al. 2003; Ravussin et al. 1986). A small chamber volume combined with a higher flow rate yields a better time constant, i.e. faster responsiveness. With a time constant of 1.53 h (without furniture), our chamber has a rather low time constant.

### Mixing time constant

The mixing time constant (Moon et al. 1995; Schoffelen et al. 1997) achieved by the air conditioning at lowest recirculation power (300 m^3^/h) was 30 times per hour or 2 min for the 9.9 m^3^ (furnished).

### Response time

**Fig. 5:**
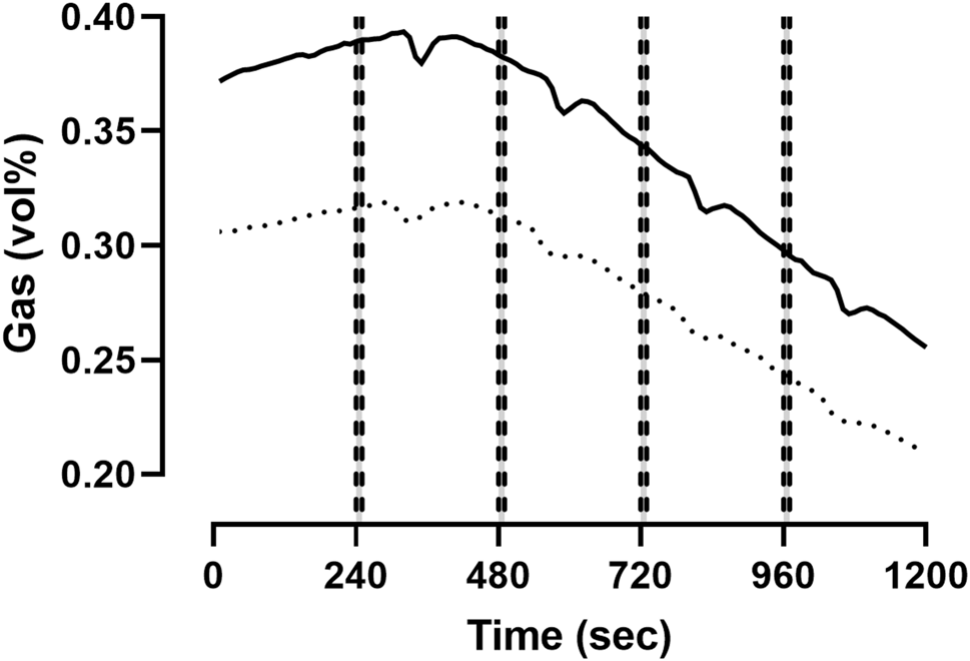
Mean response time (*n* = 6) of the chamber after opening the door four times (240, 480, 720, and 960 s; *n* = 24) for 10 s (dashed vertical lines). Oxygen (O_2_) incoming–outgoing (solid line), carbon dioxide (CO_2_) outgoing–incoming (dotted line)

To evaluate the response time of the chamber, we designed a 1200 s experiment, during which we opened the chamber door (standardized angle) four times for 10 s while acetone was being burnt inside (Fig. 5). With our usual flow rate of 120 L/min, we found responses of 0.015 ± 0.004 vol% after 80.8 ± 1.3 s for O_2_ and of 0.013 ± 0.009 vol% after 57.5 ± 2.4 s for CO_2_ (n = 24).

### Running study protocols

We start study protocols with a first, 20 min adaptation period without air flow to allow CO_2_ accumulation up to 0.3 vol%. This is followed by a second, 15 min adaption period with a flow rate of 120 L/min. Within this period, pre-specified study protocols (e.g. REE, non-exercise or exercise activity thermogenesis, dietary-induced thermogenesis) are selected from the configuration files. After adaptation, measurements start with a flow rate of 120 L/min. Depending on the protocol, the operator receives cues, such as “test meal” or “start physical activity” from the software. During measurements, a temporary safety file (*.txt) is generated. After completion, two MS Excel files are saved, one with all raw data and another with already calculated results for $\dot{V}{\text{O}}_{2}$, $\dot{V}{\text{CO}}_{{2}}$, RER, and EE.

Raw data in output files contain:

- Slope and intercept of the last calibration equations
- Factors of the last validation
- Date, time, study protocol and subject information
- Flow rate, adjusted and measured (L/min)
- Atmospheric pressure (Torr)
- Temperature inside the chamber and of incoming and outgoing air (°C)
- Relative humidity inside the chamber and of incoming and outgoing air (%)
- Raw values of O_2_ and CO_2_ of incoming and outgoing air (vol%)
- Signals of PIRS 1–3 (Volt)

Raw data are given every minute and are later analyzed with protocol specific MS Excel templates.

### Chamber resolution

**Fig. 6:**
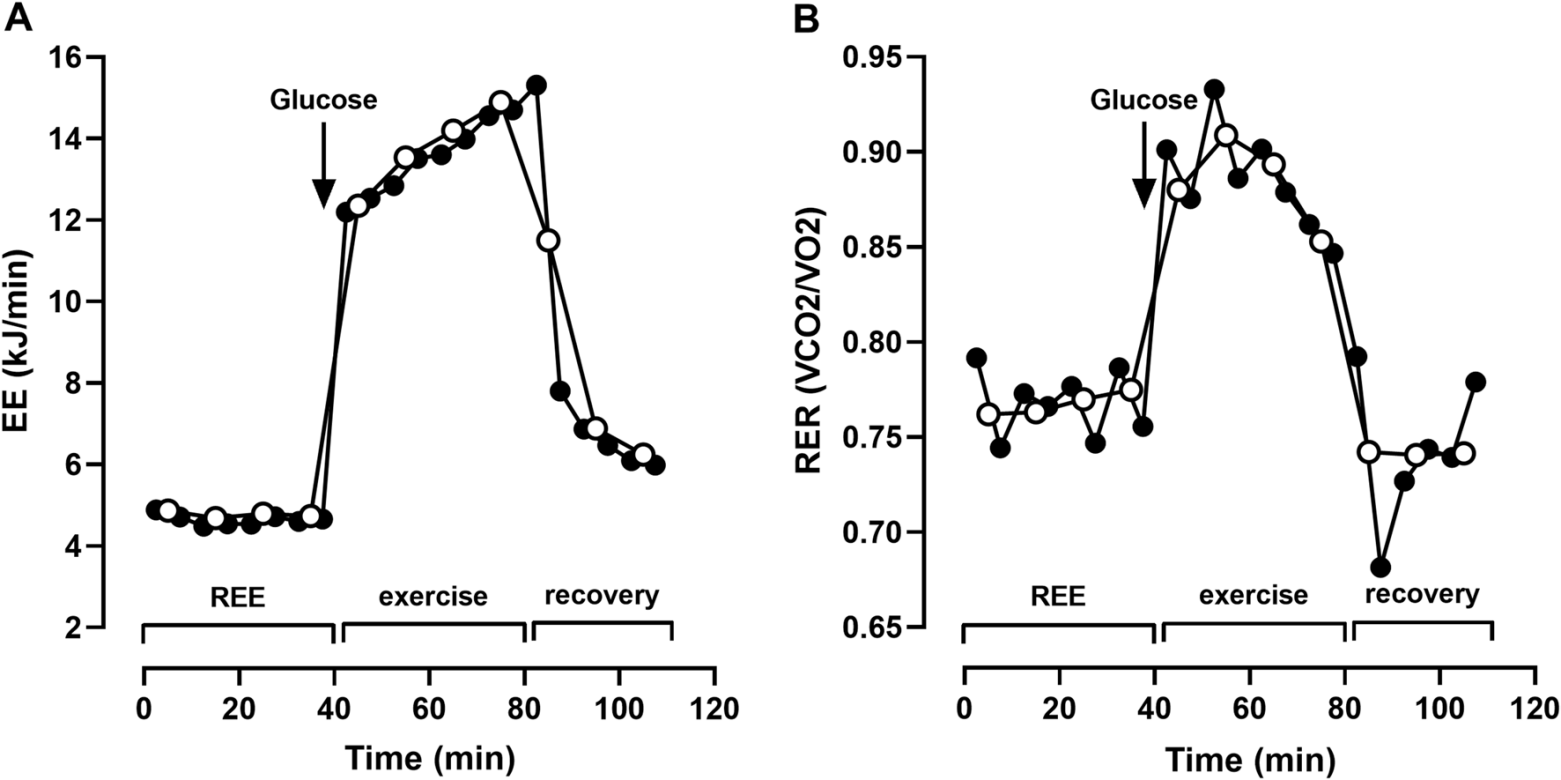
Energy expenditure (EE; **A**) and respiratory exchange ratio (RER; **B**) of 13 healthy subjects at rest (REE) and during 40 min of moderate bicycle exercise (0.5 W/kg bodyweight) followed by 30 min recovery in an armchair. Before starting to exercise, subjects drank a 75 g glucose load. Data analysis is presented in 10 min (open circles) and 5 min (closed circles) intervals to demonstrate chamber resolution

To demonstrate the high resolution of the chamber, we reanalyzed a data set of an already published clinical study (Mähler et al. 2012). This study investigated differences in energy metabolism at rest and during exercise in multiple sclerosis patients and healthy controls. For the current analysis, we used data from 13 healthy subjects. Original data were presented in 10 min intervals, i.e. averaging windows. Here, we show that there is no relevant difference between a 10 min and 5 min interval analysis of these data (Fig. 6).

### Biological reproducibility

Four repeated 60 min REE measurements of a healthy woman showed variabilities of 231.9 ± 4.8 ml/min for $\dot{V}{\text{O}}_{2}$ (CV 2.1%), 166.0 ± 6.3 ml/min for $\dot{V}{\text{CO}}_{{2}}$ (CV 3.8%), 0.73 ± 0.03 for RER (CV 4.6%), and 4.55 ± 0.07 kJ/min for EE (CV 1.6%).

## Discussion

Here, we present a precise and reliable calorimetric system with a low time constant and adequate comfort for the test subject. Since 2010, we run different study protocols with a total of 750 healthy subjects and patients with multiple sclerosis, spinocerebellar ataxia type 1, anorexia, metabolic syndrome (Haas et al. 2018; Klug et al. 2018; Mähler et al. 2012, 2014, 2015, 2018), obesity, and facioscapulohumeral muscular dystrophy (unpublished data). Our protocols include the measurement of REE, dietary-induced thermogenesis, and activity energy expenditure.

We designed the chamber to provide a non-stressful environment, with all technical equipment hidden from view. The chamber has a large window that is aligned with the window of the surrounding room, allowing a wide view outside (Fig. 1). The chamber is located on the second floor, thereby cancelling out noise and disturbances by traffic and pedestrians. The air conditioning produces very little noise and the air draft is quite moderate. Temperature can be adjusted by subjects during measurements according to their comfort.

Ideal mixing requires a mixing rate of approximately half the rooms volume per minute (Lighton 2019). Consequently, our mixing time constant can be considered optimal. To prevent air buffers, which would increase the mixing time, all furnishings have an open design to allow air to pass through.

The chamber is operated with a negative pressure of about 6 kPA in order to prevent the leakage of chamber air into the surrounding room. The air in the surrounding room, which is pulled into the chamber, is meticoulosly controlled before and during measurements. The surrounding room is large and also controlled by air conditioning to avoid ambient differences to the chamber air. In addition, there is only one operator present during measurements and he or she sits at the chamber side oposite the air inlets (about 4 m away).

For quality assurance, we meanwhile performed about 1300 calibrations (gas sensor tests) and 340 validations (2 or 20 h acetone burning tests). Oxygen and carbon dioxide sensors are calibrated before each measurement (test subject or validation) as recommended for all calorimetry systems (Schoffelen and Plasqui 2018). Sixty calibrations over 2 years indicated only minimal to small drift of the four gas analysis sensors (Fig. 2).

Correct functioning of the whole chamber is tested regularly by acetone burning. The advantage of this widely used method is the concomitant production of water vapor and heat, which closer resembles the measurement of a human than validation by gas infusion (Schoffelen and Plasqui 2018). Twenty 2 h validations over the lifetime of the chamber revealed mean deviations of 3% for $\dot{V}{\text{O}}_{2}$ and 2% for $\dot{V}{\text{CO}}_{{2}}$ (Fig. 3). Validation errors for both $\dot{V}{\text{O}}_{2}$ and $\dot{V}{\text{CO}}_{{2}}$ should be below 5%, larger deviations should initiate a search for possible causes. It is worth noting that the usage of these errors as validation factors to correct subject measurements is not optimal, because they might not be linear, i.e. they might change with differing oxygen consumption and carbon dioxide production.

We recently updated the whole measuring unit and installed two new O_2_ sensors. In addition, we currently establish new validation procedures, i.e., methanol instead of acetone combustion, because acetone may be prone to incomplete combustion. In addition, we will acquire a gas blender (HTK Hamburg, Germany) to perform gas infusion tests (Schoffelen and Plasqui 2018). These ongoing technical adaptations as well as the results of ensuing rigorous testing procedures will be reported elsewhere.

A respiration chamber can also be validated by testing biological reproducibility in healthy subjects. We found a variability of 4.55 (SD 0.07, CV 1.6%) kJ/min for 4 repeated 60 min REE measurements of a healthy women. According to different investigations by Schoffelen et al., outpatient basal metabolic rate measurements with a canopy hood show the largest variability with 4.7 (SD 0.15, CV 3.3%) kJ/min (Schoffelen and Plasqui 2018). With an SD of 0.07, biological reproducibility of our chamber was twice as high.

Compared to other chambers worldwide, whose volumes range from 10,000 to 30,000 L (Chen et al. 2020), our 11,000 L chamber is in the lower range. However, this was a deliberate choice to achieve a low time constant, i.e., response time. We are especially interested in measuring diet-induced thermogenesis and activity energy expenditure and found the fast responsiveness of our chamber to be ideal to monitor rapid changes of energy metabolism. We obtain quasi equal results if we apply 5 and 10 min intervals/averaging windows (Fig. 6). During REE measurements, when oxygen consumption is low, 10 or 15 min intervals produce less noise. However, during exercise, when oxygen consumption is high, 5 min intervals are sufficient to accurately measure activity energy expenditure. Typical modern respiration chambers require much longer intervals of 15–30 min (Schoffelen and Plasqui 2018), probably due to their larger volumes.

Although the number of operating respiration chambers has increased rather substantially to currently 40 worldwide (Chen et al. 2020), they are still quite rare in comparison to other indirect calorimetry systems (hoods, face masks). In addition to their rarity, every chamber is unique in design, technical specifications and analytical approaches. Based on the quality assurance data presented here, we conclude that our respiration chamber produced accurate, precise, valid, and stable energy expenditure measurements over an extended period of time.

## Data Availability

Additional data are available from the corresponding author on reasonable request.

## Abbreviations

EE: Energy expenditure
N_ex_: Nitrogen excretion
PIRS: Passive infrared sensors
REE: Resting energy expenditure
RER: Respiratory exchange ratio
$$\dot{V}{\text{O}}_{{2}}$$: Oxygen consumption
$\dot{V}{\text{CO}}_{2}$: Carbon dioxide production
